# Wnt Signaling Protects against Paclitaxel-Induced Spiral Ganglion Neuron Damage in the Mouse Cochlea *In Vitro*

**DOI:** 10.1155/2019/7878906

**Published:** 2019-10-07

**Authors:** Xue Wang, Yuechen Han, Man Wang, Chuan Bo, Zhenbiao Zhang, Lei Xu, Wenwen Liu, Haibo Wang

**Affiliations:** Department of Otolaryngology-Head and Neck Surgery, Shandong Provincial ENT Hospital Affiliated to Shandong University, Jinan 250022, China

## Abstract

It has been reported that paclitaxel administration could cause sensorineural hearing loss, and Wnt activation is important for the development and cell protection of mouse cochlea. However, the effect of Wnt signaling in spiral ganglion neurons (SGNs) damage induced by paclitaxel has not yet been elucidated. In this study, we explored the effect of paclitaxel on SGNs in the mouse cochlea and the neuroprotective effects of Wnt signaling pathway against paclitaxel-induced SGN damage by using Wnt agonist/antagonists *in vitro*. We first found that paclitaxel treatment resulted in a degenerative change and reduction of cell numbers in SGNs and induced caspase-mediated apoptosis in SGNs. The expression levels of *β*-catenin and C-myc were increased, thus indicating Wnt signaling was activated in SGNs after paclitaxel treatment. The activation of Wnt signaling pathway protected against SGN loss after exposure to paclitaxel, whereas the suppression of Wnt signaling in SGNs made them more vulnerable to paclitaxel treatment. We also showed that activation of Wnt signaling in SGNs inhibited caspase-mediated apoptosis. Our findings demonstrated that Wnt signaling had an important role in protecting SGNs against paclitaxel-induced damage and thus might be an effective therapeutic target for the prevention of paclitaxel-induced SGN death.

## 1. Introduction

Paclitaxel, a diterpene plant product isolated from the *Taxus chinensis*, is an effective antineoplastic agent with antimicrotubule properties. Unlike other antimicrotubule agents that induce microtubule disassembly, paclitaxel shifts the equilibrium towards microtubule assembly which is excessively stable, thereby inhibiting the dynamic reorganization of the microtubule network [1]. Paclitaxel is widely used both alone and in combination with other chemotherapeutic agents for the treatment of various carcinomas including ovarian, breast, lung, and cervical cancers as well as many head and neck neoplasms [2, 3]. Like many other antineoplastic agents, paclitaxel also exhibits many side effects of which neutropenia and peripheral neuropathy are the major dose-limiting ones [4, 5]. However, data about the effects of paclitaxel on the inner ear are very limited despite that some antineoplastic drugs have been shown to be ototoxic [6]. Recently, there have been a few publications presented a relationship between hearing loss and paclitaxel administration [7, 8], but the effect of paclitaxel on SGNs in the mouse cochlea has not yet been fully elucidated.

The Wnt signaling transduction cascade has been reported to play a central regulatory role in aspects of cell polarity, cell fate determination, cell migration, formation of the primary axis, organogenesis, and the renewal of stem cells during embryonic development [9]. Disruptions in this highly conserved and complex system lead to various pathological conditions, such as impaired bone healing, autoimmune diseases, and malignant degeneration [10]. In the mammalian inner ear, several studies have proposed that Wnt signaling also played critical roles in the otocyst induction, the formation of vestibular structures, and the cochlear development. For example, Jacques et al. reported that Wnt signaling had a dual function in controlling the proliferation and differentiation of hair cell (HC) progenitors [11]. Liu et al. showed that the activation of Wnt signaling could protect against HC damage induced by neomycin in the mouse cochlea through inhibiting the accumulation of ROS in HCs [12]. Additionally, our newly published data proved that Wnt signaling activated TP53-induced glycolysis and apoptosis regulator (TIGAR) and protected against cisplatin-induced SGN damage in the mouse cochlea [13]. Nevertheless, the effect of Wnt signaling in SGN damage induced by paclitaxel in the mouse cochlea remains unclear.

In the present study, we explored the effect of paclitaxel on SGNs in the mouse cochlea and the neuroprotective effects of Wnt signaling pathway against paclitaxel-induced SGN damage by using Wnt agonist/antagonists *in vitro*. Our findings demonstrated that the activation of Wnt signaling protected SGNs against paclitaxel-induced damage through the suppression of apoptosis in SGNs, suggesting that it might be an effective therapeutic target for the prevention of SGN damage.

## 2. Materials and Methods

### 2.1. Animals

Postnatal 3-day-old (P3) C57BL/6 mice were purchased from the Animal Center of Shandong University (Jinan, China). All experiments were approved by the Animal Care Committee of Shandong University, China, and were consistent with the National Institute of Health's Guide for the Care and Use of Laboratory Animals.

### 2.2. Cochlear Organotypic Culture and Drug Treatment

C57BL/6 mice were decapitated at P3, and only the segments isolated from the middle cochlear turn were collected to keep sampling consistence between groups. The tissue dissection procedures were carried out as described previously [13]. Briefly, the temporal bones of two sides were cut off and the cochlear capsule was removed to expose the membranous labyrinth under a dissecting microscope. The stria vascularis was removed, and the middle turn cochlear explants containing SGNs were adhered onto 10 mm glass coverslips precoated with Cell-Tak (BD Biosciences, USA). Isolated explants were cultured in 4-well dish (Greiner Bio-One, Germany) in full medium (FM) overnight at 37°C in a 5% CO_2_ atmosphere. The FM was supplemented with N_2_ (1 : 100, Invitrogen, USA), B27 (1 : 50, Invitrogen), ampicillin (50 mg/ml, Sigma, USA), epidermal growth factor (EGF, 20 ng/mL, Sigma, USA), basic fibroblast growth factor (bFGF, 10 ng/mL, Sigma), insulin-like growth factor-1 (IGF-1, 50 ng/mL, Sigma), and heparan sulfate (50 ng/mL, Sigma) in Dulbecco's modified Eagle medium/F12 (DMEM/F12, Invitrogen, USA). On the following day, the samples were treated with the following conditions as indicated in the text: fresh culture medium alone (control), fresh culture medium with various concentrations (10–30 *μ*M) of paclitaxel (EMD Millipore, USA), paclitaxel and R-spondin1 (RS-1, 10 *μ*g/mL, R&D Systems, USA), or paclitaxel and IWP-2 (10 *μ*M, Stemgent, USA). After incubation for 48 h, the samples were then used in the immunostaining and other assays.

### 2.3. Immunostaining

After organotypic culture, the explants were fixed with 4% paraformaldehyde, permeabilized with 1% Triton X-100 in PBS, and immersed in blocking solution at room temperature for 1 h. The samples were then incubated with different primary antibodies: Tuj 1 (1 : 1000, Neuromics, USA), Neu N (1 : 1000, Cell Signaling Technology, USA), cleaved-caspase 3 (1 : 400, Cell Signaling Technology, USA), PY489-*β*-catenin (1 : 400, DSHB, USA), and C-myc (1 : 800, Cell Signaling Technology, USA) diluted in blocking solution at 4°C overnight. The next day, the samples were incubated with FITC-conjugated, TRITC-conjugated, or Cy5-conjugated (1 : 1000, Invitrogen, USA) secondary antibody along with DAPI (1 : 3000, Sigma-Aldrich, USA) at room temperature for 1 h. Then, the coverslips were mounted and the samples were observed under a laser scanning confocal microscope (Leica, Germany).

### 2.4. Terminal Deoxynucleotidyl Transferase dUTP Nick-End-Labeling (TUNEL) Assay

Cell apoptosis was measured by DNA fragmentation with a TUNEL staining kit (Click-iT Plus TUNEL Assay for In Situ Apoptosis Detection, Invitrogen, USA) according to the manufacturer's instructions. Each section was stained with DAPI for 15 min at room temperature and protected from light. After washing with PBS, the samples were evaluated using the confocal microscopy (Leica, Germany).

### 2.5. RNA Extraction and Real-Time Polymerase Chain Reaction

After culturing for 48 h, total RNA was isolated from the middle turn cochlear explants using TRIzol reagent (Invitrogen, Carlsbad, CA, USA) according to the manufacturer's instructions. The cDNA was synthesized from each RNA sample by reverse transcription using the RevertAid First Strand cDNA Synthesis Kit (Thermo Scientific, USA). For analysis of *β*-catenin and C-myc, a SYBR Green PCR kit (TaKaRa, Japan) was used to quantify the mRNA levels of *β*-catenin and C-myc. GAPDH was amplified as the housekeeping gene. All data were analyzed using an Eppendorf Realplex 2. The relative expression levels of *β*-catenin and C-myc were calculated using the 2^−ΔΔCT^ method. PCR primers were *β*-catenin (forward sequence: ATGCGCTCCCCTCAGATGGTGTC; reverse sequence: TCGCGGTGGTGAGAAAGGTTGTGC), C-myc (forward sequence: GCGTTGGAAACCCCGACAG; reverse sequence: CTTCCAGATATCCTCACTGGGC), and GAPDH (forward sequence: AGGTCGGTGTGAACGGATTTG; reverse sequence: TGTAGACCATGTAGTTGAGGTCA).

### 2.6. SGN Counting

The confocal images of cultured middle turn cochlear explants were taken using a Leica confocal fluorescence microscope. Tuj 1, a neuron-specific marker that specifically labels both SGN bodies and neurites, was used for SGN staining. The number of SGNs was counted using the ImageJ software. The density of SGNs was then calculated within the unit area (0.01 mm^2^) from the middle turn sections of each cochlea.

### 2.7. Statistical Analysis

Data were presented as mean ± SD from at least three individual experiments. Two-tailed, unpaired Student's *t*-tests were used to determine statistical significance in comparisons between two groups. When comparing more than two groups, data were statistically analyzed by one-way ANOVA followed by a Dunnett multiple comparisons test. A value of *p* < 0.05 was considered to indicate a statistically significant result.

## 3. Results

### 3.1. Paclitaxel Treatment Damaged SGNs

**Figure 1:**
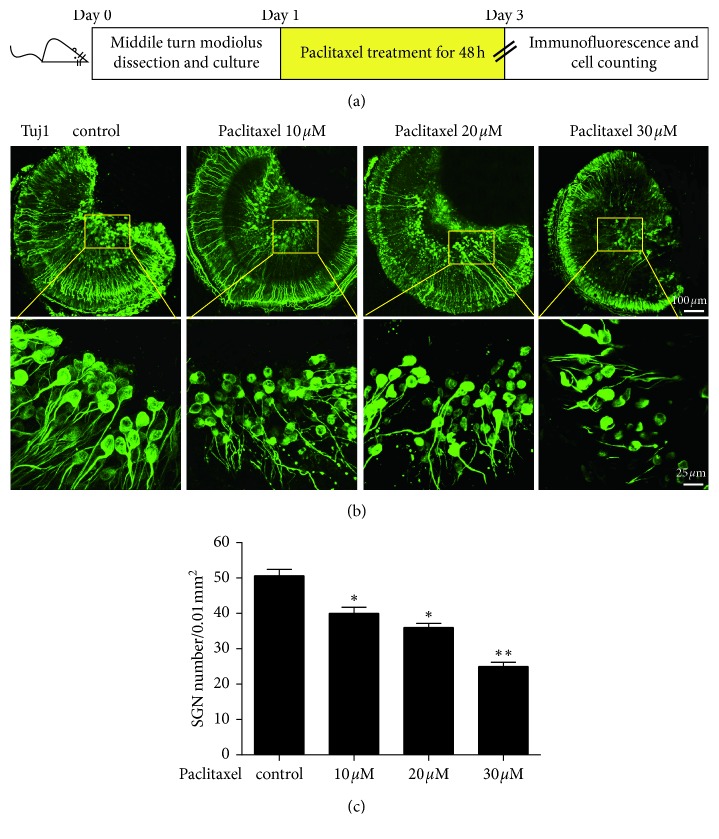
Paclitaxel treatment damaged SGNs. (a) The diagram of the assay for (b) and (c). The middle turn cochleae and SGN explants from P3 C57BL/6 WT mice were cultured and incubated with different concentrations of paclitaxel (10 *μ*M, 20 *μ*M, or 30 *μ*M) for 48 h and then used for immunostaining. (b and c) Immunofluorescence revealed paclitaxel led to a degenerative change and reduction of SGNs in a dose-dependent manner. ^*∗*^*p* < 0.05, ^*∗∗*^*p* < 0.01 vs. control group.

To examine the neurotoxic effect of paclitaxel on SGNs, the cochlea middle turn explants isolated from P3 C57BL/6 mice were treated with different concentrations of paclitaxel (10, 20, and 30 *μ*M) for 48 h, respectively (Figure 1(a)), and results showed that three concentrations of paclitaxel inhibited the survival of SGNs to varying degrees compared with the control group. As shown in Figure 1(b), in normal control groups, the soma of SGNs exhibited large, round, or oval shape and was densely packed. Immunostaining with a Tuj 1 antibody intensely labeled the cytoplasm of SGNs but weakly labeled the nucleus. In addition, the peripheral auditory nerve fibers (ANFs), which project out radially from SGNs to HCs, were also strongly labeled with Tuj 1 and organized into smooth and thick fascicles. Treatment with 10 *μ*M paclitaxel caused mild SGNs shrinkage and a slight reduction in the number of SGNs and produced slight fragments of some ANF distal ends. When treated with 20 *μ*M paclitaxel, the neurotoxic effect of paclitaxel was stronger and the mean density of SGNs was lower than in the 10 *μ*M group. After being treated with 30 *μ*M paclitaxel, there was greater shrinkage and condensation of the SGN soma, and the number of SGNs was decreased from 51 to 25 per 0.01 mm^2^ compared to the control group. In addition, the majority of ANFs were fragmented and the peripheral fiber ends approaching HCs were almost completely lost in the 30 *μ*M paclitaxel-treated group. These results indicated that paclitaxel led to a degenerative change and reduction of SGNs in a dose-dependent manner. As the treatment of 30 *μ*M paclitaxel for 48 h induced the most degenerative changes and almost 50% loss of SGNs, this condition was chosen for the SGN explant culture treatment in further experiments.

### 3.2. Paclitaxel Treatment Induced Caspase-Mediated Apoptosis in SGNs

**Figure 2:**
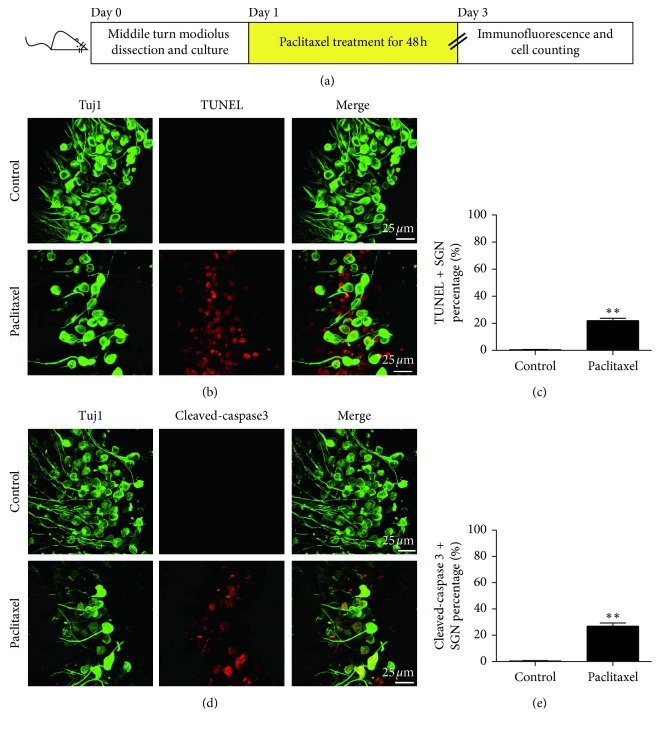
Paclitaxel treatment induced caspase-mediated apoptosis in SGNs. (a) The diagram of the assay for (b)–(e). The middle turn cochleae and SGN explants from P3 C57BL/6 WT mice were cultured and incubated with 30 *μ*M paclitaxel for 48 h and then used for immunostaining. (b) Representative confocal images of SGNs labeled with DAPI (grey), Tuj1 (green), and TUNEL (red). (c) Statistical data showed that TUNEL/Tuj1 double-positive cells were significantly increased in the paclitaxel-treated group. (d) Representative confocal images of cleaved-caspase 3 (red) and Tuj1 (green) immunofluorescence. (e) Statistical data showed that the cleaved-caspase 3/Tuj1 double-positive SGNs in the paclitaxel-treated group were statistically increased compared to the control group. ^*∗∗*^*p* < 0.01 vs. control group.

To determine whether the paclitaxel-induced cell death of SGNs was mediated by apoptosis, TUNEL staining of cochlear SGNs in the middle turn was examined following treatment in the absence or presence of paclitaxel (30 *μ*M) for 48 h (Figure 2(a)). The SGNs that were double labeled by Tuj 1 (green fluorescence) and TUNEL (red fluorescence) in the nuclei were considered to be apoptotic SGNs. The results showed that no double-positive SGNs were detected in the normal control explants, while a significant increase in the numbers of apoptotic SGNs was observed after treatment with paclitaxel (*p* < 0.01) (Figures 2(b) and 2(c)). Furthermore, the expression of cleaved-caspase 3 was also examined by immunostaining to evaluate whether paclitaxel-induced SGN apoptosis was mediated by caspase. As shown in Figures 2(d) and 2(e), the double-positive SGNs labeled by Tuj 1 (green fluorescence) and cleaved-caspase 3 (red fluorescence) were not detected in SGNs in control cultures. However, after being treated with 30 *μ*M paclitaxel for 48 h, there were significantly more cleaved-caspase 3/Tuj1 double-positive SGNs (*p* < 0.01), suggesting that caspase 3 was activated in SGNs after paclitaxel treatment. Taken together, these data indicated that paclitaxel-induced cell death of SGNs was mediated by apoptosis in a caspase-dependent manner.

### 3.3. Wnt Signaling Was Activated in SGNs after Paclitaxel Treatment

**Figure 3:**
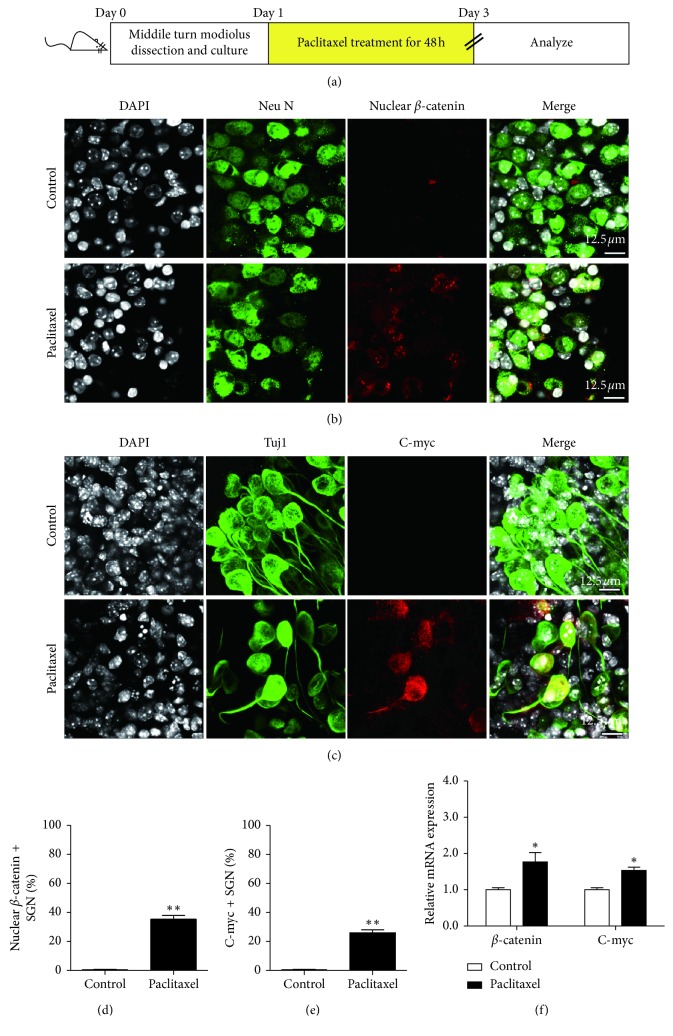
Wnt signaling was activated in SGNs after paclitaxel treatment. (a) The diagram of the assay for (b)–(f). The SGN explants from P3 C57BL/6 WT mice were cultured and incubated with 30 *μ*M paclitaxel for 48 h and then used for immunostaining and qPCR. (b) Representative confocal images of SGNs labeled with DAPI (grey), Neu N (green), and nuclear *β*-catenin (red). (c) Representative confocal images of SGNs labeled with DAPI (grey), Tuj1 (green), and C-myc (red). (d and e) Statistical data showed that the nuclear *β*-catenin/Neu N and C-myc/Tuj1 double-positive SGNs in the paclitaxel-treated group were statistically increased compared to the control group. (f) qPCR showed that the mRNA expression of *β*-catenin and C-myc was significantly upregulated after paclitaxel treatment. ^*∗*^*p* < 0.05, ^*∗∗*^*p* < 0.01 vs. control group.

Furthermore, to determine whether Wnt signaling also plays a role in against SGN damage in the mouse cochlea induced by paclitaxel, we investigated whether Wnt signaling was activated in SGNs after paclitaxel treatment. *β*-Catenin is the key factor in the canonical Wnt signaling pathway, which migrates to the nucleus upon Wnt activation to activate the expression of Wnt target genes. C-myc is a key transcriptional target of the Wnt signaling pathway and has been reported to mediate many cellular responses to Wnt signaling [14, 15]. Therefore, to evaluate the activation of the Wnt signaling pathway, the expressions of *β*-catenin and Wnt target gene C-myc in SGNs after treatment with 30 *μ*M paclitaxel for 48 h were measured by immunostaining and qRT-PCR, respectively (Figure 3(a)). As illustrated in Figures 3(b) and 3(e), the protein expressions of nuclear *β*-catenin and C-myc in SGNs were almost undetectable in the control group, while their expressions were significantly upregulated in the SGNs after paclitaxel injury (*p* < 0.01). qRT-PCR result also revealed that the mRNA expressions of *β*-catenin and C-myc were significantly increased by treatment with paclitaxel (Figure 3(f)) (*p* < 0.05).

Nevertheless, the results seem to be discrepancy as paclitaxel induces SGN loss significantly while it also activates the Wnt signaling pathway. One hypothesis to explain this might be that the lower doses of paclitaxel activate the self-repair system of the cochleae via activating Wnt signaling, thus producing a self-healing injury. To test this hypothesis, the effect of low concentrations of paclitaxel (1 and 5 *μ*M) on cultured SGNs was evaluated. The middle turn cochlear explants were cultured and treated with 1 or 5 *μ*M paclitaxel for 48 h, respectively (Supplementary Figure 1(a)). As illustrated in Supplementary [Supplementary-material supplementary-material-1], although statistical analysis showed there was no significant difference in SGN numbers between the control group and paclitaxel-treated groups (Supplementary Figure 1(b)), the nuclear expression of *β*-catenin was detected in both the 1 *μ*M and 5 *μ*M paclitaxel-treated groups (Supplementary Figure 1(c)), revealing that low-dose paclitaxel treatment also activated Wnt signaling in SGNs which are spared apoptosis. Taken together, these results demonstrated that the Wnt signaling was activated in the cochlear SGNs after paclitaxel treatment, and thus, it might play a role in protecting SGNs against paclitaxel damage.

### 3.4. Activation of Wnt Signaling Protected SGNs against Paclitaxel-Induced Damage

**Figure 4:**
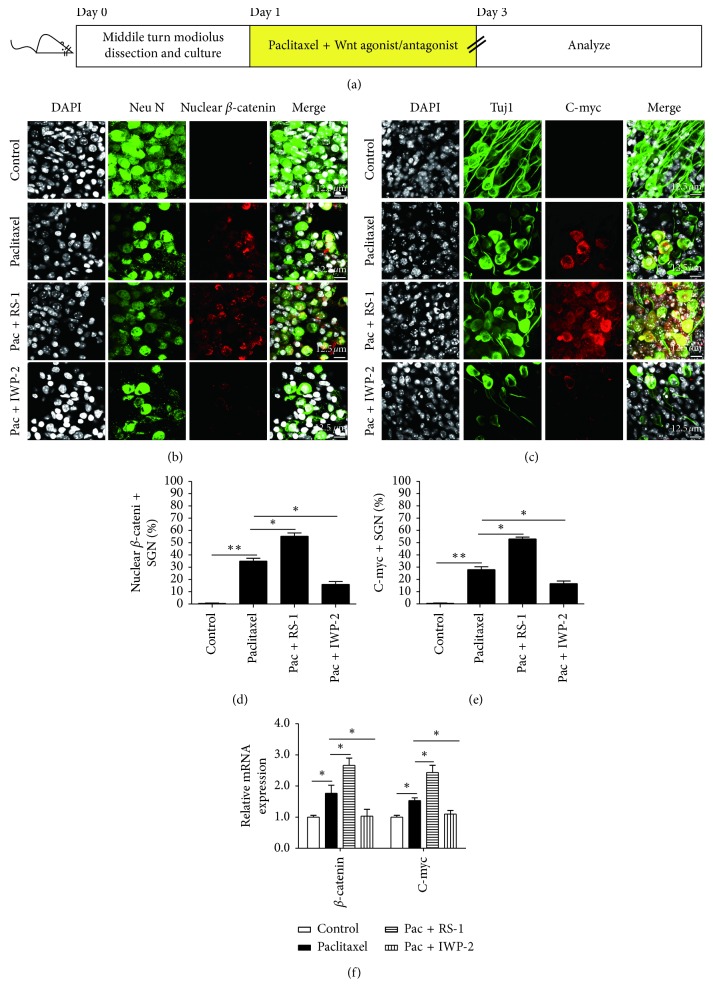
Inhibition and activation of Wnt signaling affected *β*-catenin and C-myc expression in SGNs after paclitaxel treatment. (a) The diagram of the assay for (b)–(f). The middle turn cochlear SGN explants from P3 mice were cultured *in vitro*, and the explants were treated with 30 *μ*M paclitaxel, 30 *μ*M paclitaxel together with a Wnt agonist (10 *μ*g/ml RS-1), or 30 *μ*M paclitaxel together with a Wnt antagonist (10 *μ*M IWP-2) for 48 h and then used for immunostaining and qPCR. (b–e) Immunofluorescence revealed the treatment with RS-1 resulted in an increase in the protein expression of *β*-catenin and C-myc in SGNs, while treatment with IWP-2 markedly reduced the expression of *β*-catenin and C-myc in SGNs compared with the paclitaxel-only group. (f) qPCR showed that the mRNA expression of *β*-catenin and C-myc was significantly upregulated after treatment with RS-1. ^*∗*^*p* < 0.05, ^*∗∗*^*p* < 0.01.

**Figure 5:**
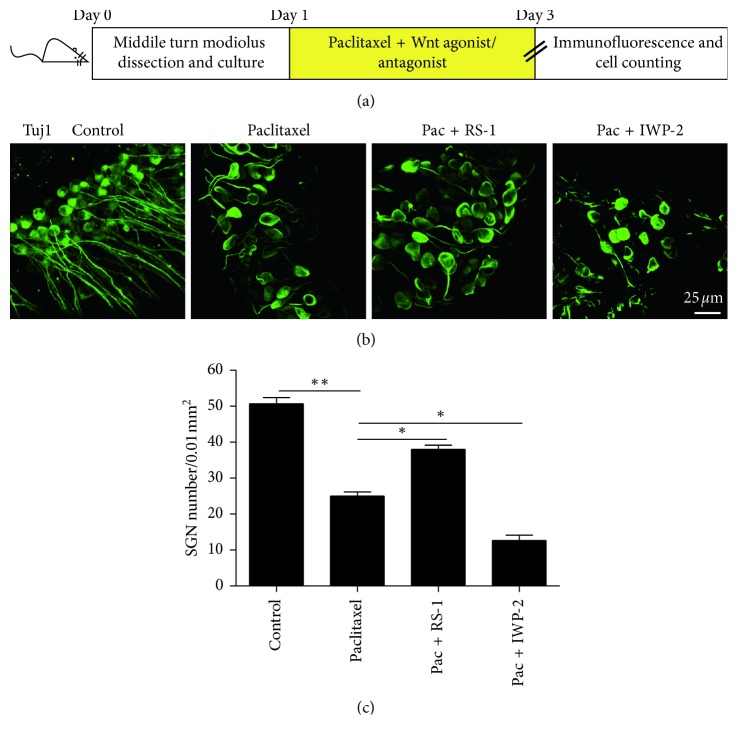
Wnt signaling promoted SGN survival after paclitaxel-induced damage. (a) The diagram of the assay for (b) and (c). The middle-turn cochlear SGN explants from P3 WT mice were treated with 30 *μ*M paclitaxel, 30 *μ*M paclitaxel together with a Wnt agonist (10 *μ*g/ml RS-1), or 30 *μ*M paclitaxel together with a Wnt antagonist (10 *μ*M IWP-2) for 48 h. (b and c) Tuj 1 staining (green) showed that the Pac + RS-1 group had significantly more surviving SGNs than the paclitaxel-only group, while in the Pac + IWP-2 group there were significantly fewer surviving SGNs compared to the paclitaxel-only group. ^*∗*^*p* < 0.05, ^*∗∗*^*p* < 0.01.

Then, in order to investigate the effect of Wnt signaling on paclitaxel-induced SGN damage, we used the Wnt agonists (RS-1) and Wnt antagonist (IWP-2) to activate or suppress Wnt signaling in SGNs with paclitaxel treatment. In the experiments, the middle turn cochlear SGN explants from P3 mice were treated with 30 *μ*M paclitaxel, 30 *μ*M paclitaxel together with RS-1 (10 *μ*g/ml), or 30 *μ*M paclitaxel together with IWP-2 (10 *μ*M) for 48 h, respectively (Figures 4(a) and 5(a)). We first confirmed that the expression levels of *β*-catenin and C-myc were indeed affected by the Wnt agonist and antagonist, and immunostaining and qRT-PCR assays were conducted to show the protein and mRNA expressions of *β*-catenin and C-myc, respectively. We found that the treatment with RS-1 resulted in an increase in both the protein and mRNA expressions of *β*-catenin and C-myc in SGNs, while treatment with IWP-2 markedly reduced the expressions of *β*-catenin and C-myc in SGNs compared with the paclitaxel-only group (*p* < 0.05 or *p* < 0.01) (Figures 4(b) and 4(f)). We further assessed the effects of increased or decreased Wnt signaling on SGN survival by counting the Tuj 1-positive SGN number in 0.01 mm^2^ in the middle turn of the cochlea after paclitaxel damage. Tuj 1 staining showed that the Pac + RS-1 group had significantly more surviving SGNs compared to the paclitaxel-treated group. However, significantly fewer surviving SGNs were detected when treated with paclitaxel in the presence of IWP-2, compared to the paclitaxel-treated group (*p* < 0.05) (Figures 5(b) and 5(c)). Moreover, in order to clarify the role of RS-1 or IWP-2 itself on SGNs, we also detected the RS-1-only and IWP-2-only controls. As illustrated in Supplementary [Supplementary-material supplementary-material-1], cochlear middle turn explants from P3 C57BL/6 mice were cultured with RS-1 (10 *μ*g/ml) or IWP-2 (10 *μ*M) without paclitaxel treatment for 48 h, respectively (Supplementary [Supplementary-material supplementary-material-1]). Immunofluorescence results demonstrated that nuclear expression of *β*-catenin and upregulation of C-myc were detected in RS-1-treated SGNs but not in the IWP-2 alone group (Supplementary Figures [Supplementary-material supplementary-material-1] and [Supplementary-material supplementary-material-1]). However, there was no significant difference in SGN number no matter in the RS-1-only or IWP-2-only group compared to the control group (Supplementary [Supplementary-material supplementary-material-1]), which indicated that the activation or inhibition of Wnt signaling in normal SGNs would not impact the survival of SGNs in our study, and the increased SGN loss could not be induced by IWP-2 alone. Taken together, all of these results suggested that the activation of Wnt signaling could protect SGNs against paclitaxel-induced damage.

### 3.5. Wnt Signaling Regulated the Caspase-Mediated Apoptosis in SGNs as a Result of Paclitaxel Treatment

**Figure 6:**
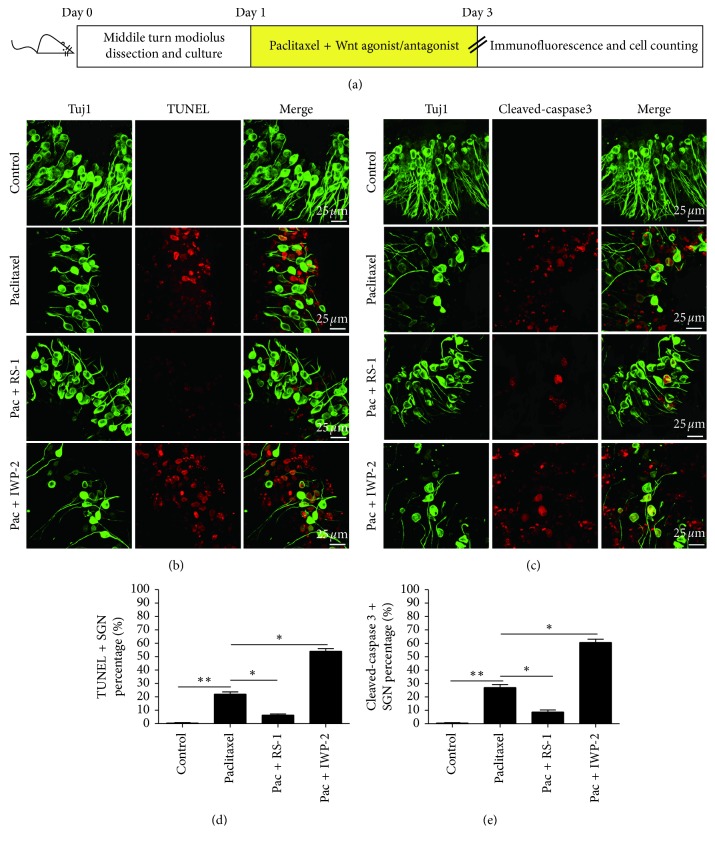
Wnt signaling regulated the caspase-mediated apoptosis in SGNs as a result of paclitaxel treatment. (a) The diagram of the assay for (b)–(e). The cochlear SGN explants from P3 mice were treated with 30 *μ*M paclitaxel, 30 *μ*M paclitaxel together with a Wnt agonist (10 *μ*g/ml RS-1), or 30 *μ*M paclitaxel together with a Wnt antagonist (10 *μ*M IWP-2) for 48 h and then used for immunostaining. (b–e) After paclitaxel treatment, the SGN explants treated with paclitaxel and RS-1 had significantly fewer TUNEL/Tuj 1 and cleaved-caspase 3/Tuj 1 double-positive SGNs compared to the paclitaxel-only group, whereas the paclitaxel and IWP-2 cotreated cochleae had significantly more TUNEL/Tuj 1 and cleaved-caspase 3/Tuj 1 double-positive SGNs. ^*∗*^*p* < 0.05, ^*∗∗*^*p* < 0.01.

We further investigated the effect of Wnt signaling on the caspase-mediated apoptosis of SGNs induced by paclitaxel. Cochlear SGN explants in the middle turn were treated with 30 *μ*M paclitaxel alone, 30 *μ*M paclitaxel together with a Wnt agonist (10 *μ*g/ml RS-1), or 30 *μ*M paclitaxel together with a Wnt antagonist (10 *μ*M IWP-2) for 48 h, respectively. Then, TUNEL and cleaved-caspase 3 staining in SGNs were examined (Figure 6(a)). Our results showed that the paclitaxel together with RS-1 group had significantly fewer TUNEL/Tuj 1 double-positive SGNs and cleaved-caspase 3/Tuj 1 double-positive SGNs compared with the paclitaxel-treated group, while SGN explants treated with paclitaxel together with IWP-2 had significantly more TUNEL/Tuj 1 double-positive and cleaved-caspase 3/Tuj 1 double-positive SGNs in the middle turns (*p* < 0.05) (Figures 6(b) and 6(e)). Therefore, these results demonstrated that the activation of Wnt signaling could inhibit the caspase-mediated apoptosis in SGNs as a result of paclitaxel treatment.

## 4. Discussion

It is well known that paclitaxel is used widely for the treatment of various cancers and cardiovascular diseases, and peripheral neuropathy is the most important nonhematological adverse effect of paclitaxel therapy [16]. However, the data with respect to paclitaxel-induced ototoxicity are very limited. Recently, paclitaxel has been reported to cause mild to moderate sensorineural hearing loss and some histopathologic changes in the mouse cochlea, and paclitaxel can damage cochlear HCs, ANFs, and SGNs near the base of the cochlea [8]. Here, in order to examine the neurotoxic effect of paclitaxel on SGNs, middle cochlear turn explants isolated from P3 mice were treated with different concentrations of paclitaxel (10, 20, and 30 *μ*M) for 48 h. Our results demonstrated that SGNs underwent a degenerative change and reduction of cell numbers in response to paclitaxel treatment in a dose-dependent manner *in vitro*.

Identification of the pathways promoting cell injury which leads to cell death is the key to understand mechanisms of paclitaxel promoting degeneration of the SGNs in the inner ear. Paclitaxel is well known to exert antitumor activities in a variety of tumor cells through induction of apoptosis, such as breast, bone, and solid tumor [17–19]. Additionally, it has also been reported that paclitaxel can also induce apoptosis in cortical neurons by regulating JNK activity and its downstream transcription and inhibition of the PI3K/AKT pathway [20]. In the present study, to determine whether the paclitaxel-induced cell death of SGNs was also mediated by apoptosis, TUNEL staining of SGNs in the middle turn was examined following treatment with paclitaxel. We found that paclitaxel treatment resulted in positive TUNEL staining in the SGNs indicative of an apoptotic mechanism. Caspase activation, and specifically that of caspase 3, could be considered as the hallmark of intrinsic apoptosis. Caspase 3-mediated apoptosis pathway has been reported to appear pivotal to medicine-originated ototoxicity, and members of the intrinsic apoptosis caspase cascade are activated after ototoxic medicine treatment [21–23]. Consistent with these previous findings, our results showed that there were significantly more cleaved-caspase 3/Tuj1 double-positive SGNs in the paclitaxel-treated group, suggesting that caspase 3 was activated in SGNs after paclitaxel treatment. Taken together, these data indicated that paclitaxel treatment induced caspase-mediated apoptosis in SGNs.

Numerous studies have demonstrated that Wnt signaling is an important signaling pathway which is associated with many physiological functions, including cell survival, proliferation, and cellular protection [24, 25]. The canonical Wnt signaling pathway has been shown to be important for the development of the mouse cochlea, including HC regeneration and cell differentiation [26]. In addition, canonical Wnt signaling has also been reported to protect HCs and SGNs against damage induced by ototoxic medicine, including neomycin and cisplatin in the mouse cochlea in recent years [12, 13]. Here, in order to study whether canonical Wnt signaling was related to protecting SGNs against paclitaxel-induced damage in SGNs in the mouse cochlea, we explored whether Wnt signaling was activated in SGNs after paclitaxel treatment. It has been reported that the canonical Wnt signaling is activated when secreted Wnts bind Frizzled receptors, causing downregulation of glycogen synthase kinase 3*β* activity and subsequent stabilization and nuclear translocation of cytoplasmic *β*-catenin. Within the nucleus, stabilized *β*-catenin associates with TCF/Lef transcription factors to activate target genes [27]. C-myc, a downstream target of canonical Wnt/*β*-catenin signaling, functions as a regulator of many cellular responses to Wnt/*β*-catenin signaling. For example, Wong et al. reported that Wnt signaling pathway played a critical role in naive T-cell survival through regulating C-myc [28]. He et al. reported that Wnt/*β*-catenin signaling pathway could upregulate C-myc expression to promote pancreatic stem cell proliferation and made more cells survive in autoimmune patients [29]. In this study, results showed that paclitaxel (10, 20, and 30 *μ*M) exposure induced SGN loss as well as Wnt signaling activation in SGNs, with the increase of both protein and mRNA expressions of *β*-catenin and C-myc in the paclitaxel-treated group (Figure 3). Interestingly, the activation of Wnt signaling was also observed in SGNs under lower concentrations (1 and 5 *μ*M) of paclitaxel treatment, which caused no significant SGN loss (Supplementary [Supplementary-material supplementary-material-1]). Taken together, we assume that paclitaxel might produce a self-healing injury in SGNs via activating Wnt signaling under the low-dose (1 and 5 *μ*M) treatment condition; however, when it comes to high-dose (10, 20, and 30 *μ*M) treatment, this self-protection effect seems insufficient to prevent the paclitaxel-induced damage, and thus, it might need to be augmented in order to effectively resist the paclitaxel insult.

Furthermore, we used Wnt agonist and antagonists to thoroughly examine the effect of canonical Wnt signaling on SGNs after exposure to paclitaxel. We found that the treatment with Wnt agonist RS-1 resulted in an increase in both the protein and mRNA expressions of *β*-catenin and C-myc in SGNs, while treatment with Wnt antagonist IWP-2 markedly reduced the expressions of *β*-catenin and C-myc in SGNs compared with the paclitaxel-only group. In addition, Wnt signaling protected against paclitaxel-induced SGN loss and that inhibition of Wnt/β-catenin made the SGNs more sensitive to paclitaxel-induced damage.

We further examined the mechanisms underlying the protection provided by Wnt signaling against paclitaxel-induced SGN damage, and results showed that activation of Wnt/*β*-catenin signaling in SGNs inhibited caspase-mediated SGNs apoptosis after paclitaxel treatment and that inhibition of Wnt/*β*-catenin signaling increased caspase-mediated SGN apoptosis after paclitaxel treatment, which was consistent with previous findings. Studies have proposed that Wnt/*β*-catenin has a protective function against apoptosis in many different organs and cell lines. For instance, Dehner et al. reported that Wnt/*β*-catenin negatively regulated the proapoptotic transcription factor Foxo3 and inhibited Foxo3-induced apoptosis in human HCT116 colon cancer cells [30]. Tao et al. showed that Wnt/*β*-catenin protected against hepatotoxin 3,5-diethoxycarbonyl-1,4-dihydrocollidine (DDC)-induced liver injury and inhibited oxidative stress-induced apoptosis in the liver [31]. More importantly, the effect of Wnt/*β*-catenin in protecting inner ear cells against caspase-dependent apoptosis has been reported recently. Kim et al. declared that, in the rat sensory epithelium OC1 cell line, Wnt/*β*-catenin protected the OC1 cells against cisplatin-induced cell death [32]. Liu et al. showed that activating Wnt signaling protected against HC damage induced by neomycin through reducing caspase-dependent apoptosis [12], and the newly published data of our group demonstrated that the activation of Wnt signaling and TIGAR protected against cisplatin-induced SGNs injury by inhibiting the accumulation of ROS in SGNs and the subsequent suppression of SGN apoptosis [13]. Thus, the data presented in this study supplemented the role of Wnt signaling pathway in protecting against inner ear injury; however, the mechanisms underlying how Wnt/*β*-catenin signaling regulate its target genes, including C-myc, to play the protective role are still unclear and need to be further explored.

## 5. Conclusions

In summary, we revealed in this study that paclitaxel treatment resulted in a degenerative change and reduction of cell numbers in SGNs and induced caspase-mediated apoptosis of SGNs. Wnt signaling was activated in SGNs after paclitaxel treatment, and the activation of Wnt signaling pathway protected against SGN loss after exposure to paclitaxel, whereas the suppression of Wnt signaling in SGNs made them more vulnerable to paclitaxel treatment. We also demonstrated that activation of Wnt signaling in SGNs inhibited caspase-mediated apoptosis. Taken together, our findings indicated that Wnt signaling was essential for protecting SGNs from paclitaxel-induced damage and thus might be an effective therapeutic target for the prevention of paclitaxel-induced SGN death.

## Data Availability

The immunostaining and qPCR data used to support the findings of this study are available from the corresponding author upon request.

## Supplementary Materials

Supplementary Materials Supplementary Figure 1: the effect of low-concentration paclitaxel on SGNs. (a) Diagram of the assay for (b) and (c). The middle turn cochleae and SGN explants from P3 C57BL/6 WT mice were cultured and incubated with 1 *μ*M or 5 *μ*M paclitaxel for 48 h and then used for immunostaining analysis. (b) There was no significant difference in SGN numbers between the control group and paclitaxel-treated groups. (c) The nuclear expression of *β*-catenin was detected in both the 1 *μ*M and 5 *μ*M paclitaxel-treated groups. Supplementary Figure 2: the effect of Wnt agonist and antagonist on SGNs. (a) The diagram of the assay for (b)–(d). The middle turn cochlear SGN explants from P3 C57BL/6 mice were cultured with a Wnt agonist (10 *μ*g/ml RS-1) or a Wnt antagonist (10 *μ*M IWP-2) without paclitaxel treatment for 48 h and then used for immunostaining. (b) Immunofluorescence demonstrated there was no significant difference in SGN number no matter in the RS-1-only or IWP-2-only group compared to the control group. (c-d) Nuclear expression of *β*-catenin and upregulation of C-myc were detected in RS-1 treated SGNs but not in the IWP-2 alone group.
