# Supplementary figures and images for: Wnt Signaling Protects against Paclitaxel-Induced Spiral Ganglion Neuron Damage in the Mouse Cochlea In Vitro
Source: Biomed Res Int. 2019 Oct 7;2019:7878906. doi: 10.1155/2019/7878906 (PMC6800971; doi:10.1155/2019/7878906)

Supplementary Figure 1

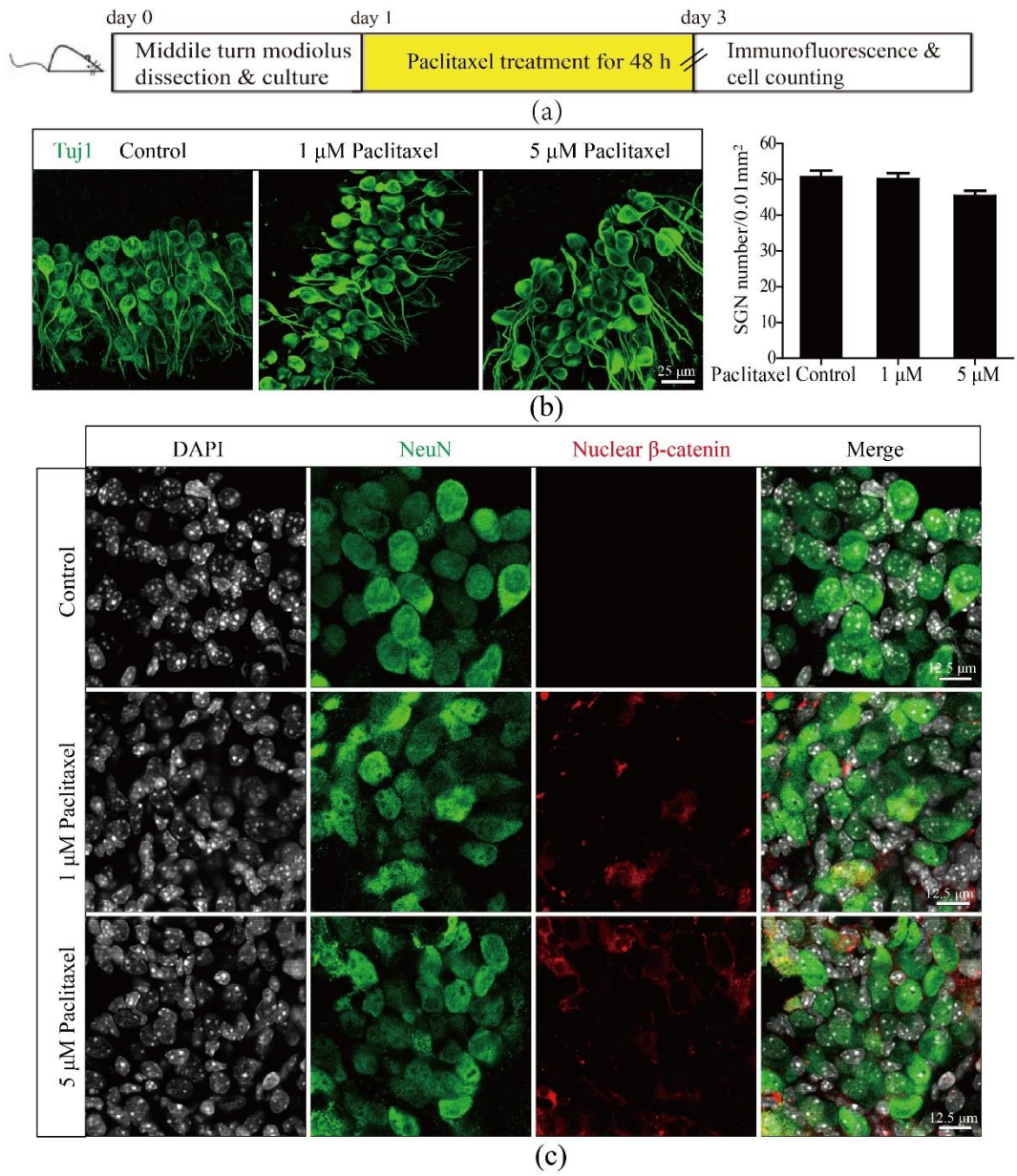

Supplementary Figure 2

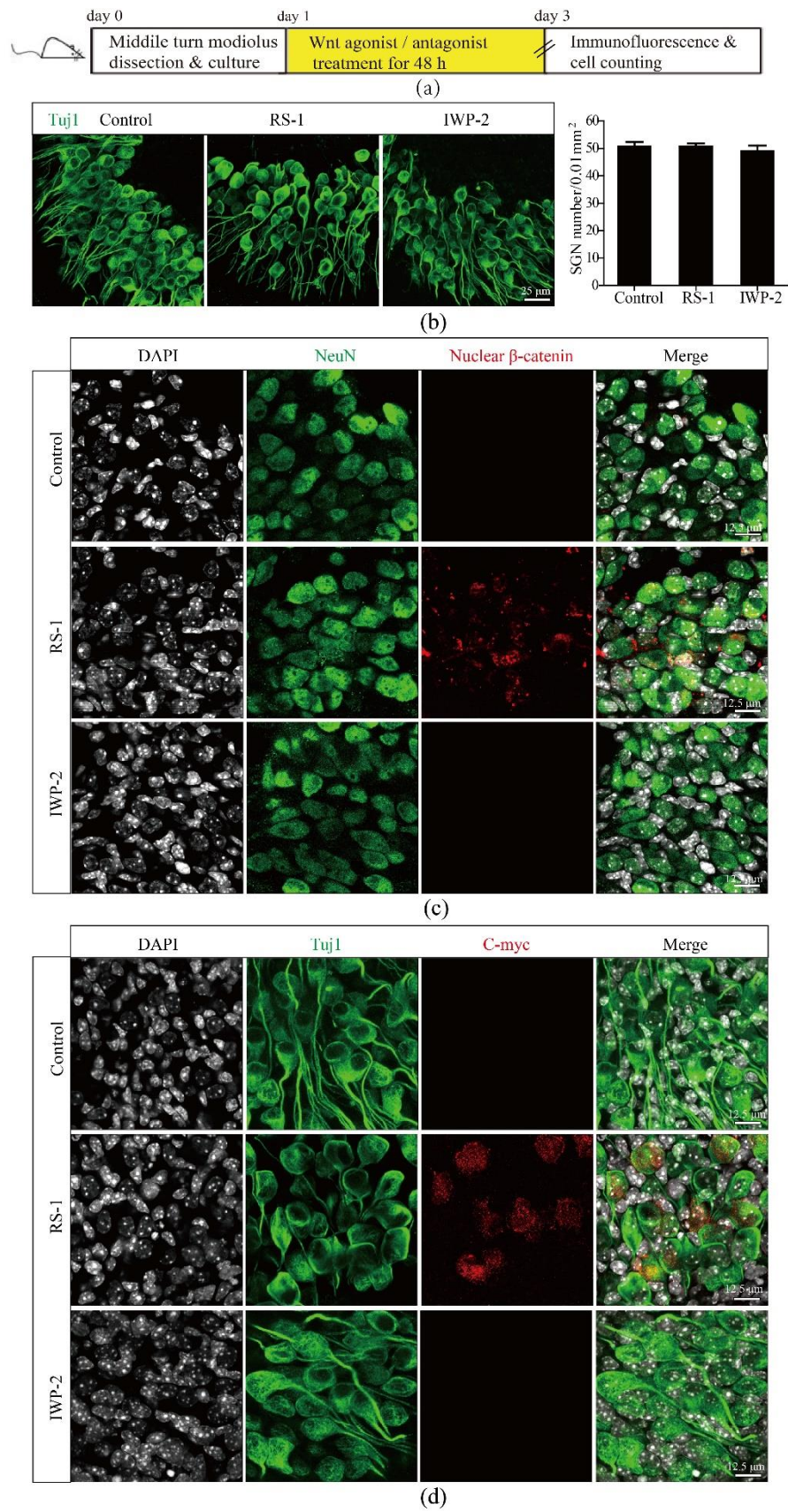

Supplement: Supplementary Materials — Supplementary Figure 1: the effect of low-concentration paclitaxel on SGNs. (a) Diagram of the assay for (b) and (c). The middle turn cochleae and SGN explants from P3 C57BL/6 WT mice were cultured and incubated with 1 μM or 5 μM paclitaxel for 48 h and then used for immunostaining analysis. (b) There was no significant difference in SGN numbers between the control group and paclitaxel-treated groups. (c) The nuclear expression of β-catenin was detected in both the 1 μM and 5 μM paclitaxel-treated groups. Supplementary Figure 2: the effect of Wnt agonist and antagonist on SGNs. (a) The diagram of the assay for (b)–(d). The middle turn cochlear SGN explants from P3 C57BL/6 mice were cultured with a Wnt agonist (10 μg/ml RS-1) or a Wnt antagonist (10 μM IWP-2) without paclitaxel treatment for 48 h and then used for immunostaining. (b) Immunofluorescence demonstrated there was no significant difference in SGN number no matter in the RS-1-only or IWP-2-only group compared to the control group. (c-d) Nuclear expression of β-catenin and upregulation of C-myc were detected in RS-1 treated SGNs but not in the IWP-2 alone group. [file 7878906.f1.pdf]
